# Semantic Framework for Social Robot Self-Configuration

**DOI:** 10.3390/s130607004

**Published:** 2013-05-28

**Authors:** Gorka Azkune, Pablo Orduña, Xabier Laiseca, Eduardo Castillejo, Diego López-de-Ipiña, Miguel Loitxate, Jon Azpiazu

**Affiliations:** 1 Tecnalia—Industry and Transport Division, Paseo Mikeletegi 7, Donostia E-20009, Spain; E-Mails: miguel.loichate@tecnalia.com (M.L.); jon.azpiazu@tecnalia.com (J.A.); 2 DeustoTech—Deusto Institute of Technology, University of Deusto, Avda Universidades 24, Bilbao E-48007, Spain; E-Mails: pablo.orduna@deusto.es (P.O.); xabier.laiseca@deusto.es (X.L.); eduardo.castillejo@deusto.es (E.C.); dipina@deusto.es (D.L.-I.)

**Keywords:** self-configuration, ontologies, social robots, healthcare environments

## Abstract

Healthcare environments, as many other real world environments, present many changing and unpredictable situations. In order to use a social robot in such an environment, the robot has to be prepared to deal with all the changing situations. This paper presents a robot self-configuration approach to overcome suitably the commented problems. The approach is based on the integration of a semantic framework, where a reasoner can take decisions about the configuration of robot services and resources. An ontology has been designed to model the robot and the relevant context information. Besides rules are used to encode human knowledge and serve as policies for the reasoner. The approach has been successfully implemented in a mobile robot, which showed to be more capable of solving situations not pre-designed.

## 1. Introduction

As the proportion of the elderly people keeps increasing, programs such as Ambient Assisted Living http://www.aal-europe.eu/ are being developed to enhance their quality of life taking advantage of the latest ICT technologies. The employed technologies and targeted situations in the literature are diverse [1–3], but they all attempt to reduce healthcare costs by providing a more independent life to patients through ICT based solutions.

In this line, the use of robotics, in particular social robotics, given the satisfying human-robot interaction [4], is being studied to achieve these target situations, both at health center [5] and at patients' home [6]. As Section 2 of this contribution details, the motivation of this research is indeed based on a real experiment developed as part of the ACROSS http://www.acrosspse.com/ project, already published in the literature [7,8], where a robot assisted healthcare providers in a health center by interacting with the patient and archiving the measurements taken to enable future analysis by therapists. While the use case provides the motivation and a clear scenario, its final application is outside the scope of this contribution.

On this experiment, certain limitations arose during the deployment. Robots in general need to be aware of their context in order to perform tasks more effectively. However, social robots involved in a health environment, whether they are at the patients' home or at the health center, need to obey especially complex rules that may change with time and even depend on specific patients. Therefore, a flexible solution to enable the robot to configure itself with complex and dynamic rules is particularly necessary in these environments.

The focus of this contribution is to explore a self-configuration solution for social robotics that enables their integration in health scenarios. In Section 2, the initial scenario from which the contribution starts will be described, while the analysis of the scenario itself is beyond the scope of this paper. The proposed solution is described in Section 3, outlining the developed techniques. The evaluation of the presented work is done in Section 4, where experimental data is discussed. Section 5 shows the related work and finally Section 6 summarizes the outcomes and the proposed solution, pointing out possible future works.

## 2. Initial Scenario

As part of the ACROSS project, a joint effort between the ABAT hospital, M-Bot Solutions and the University of Deusto was developed to assist therapists [7,8]. This scenario is not going to be presented in detail since the focus is to provide certain context to explain the solution discussed in this contribution.

**Figure 1.**
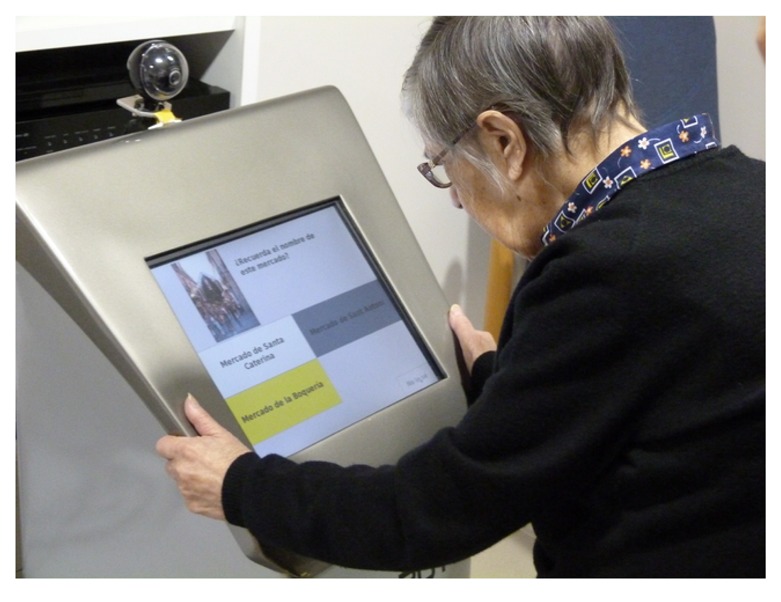
Patient using health app in the ACROSS project [7].

The scenario was divided into two applications, both running in the robot. The first application was used by the patients themselves: the robot moved to the user and it showed a set of customized questions so as to check that the patient recalled correctly places where she had been living, pictures of her family, music that she might remember and so forth. These questions were prepared by therapists, who additionally tagged the questions (e.g., visual, personal). Figure 1 shows the patient answering the robot. The robot will store in a Triple Space [9] information such as what questions were answered, how long it took the patient in answering, or which questions were not answered. The second application consumed the information stored by the first application, so therapists could see the results of the patient and the historic evolution in the quality of the responses for each tag.

**Figure 2.**
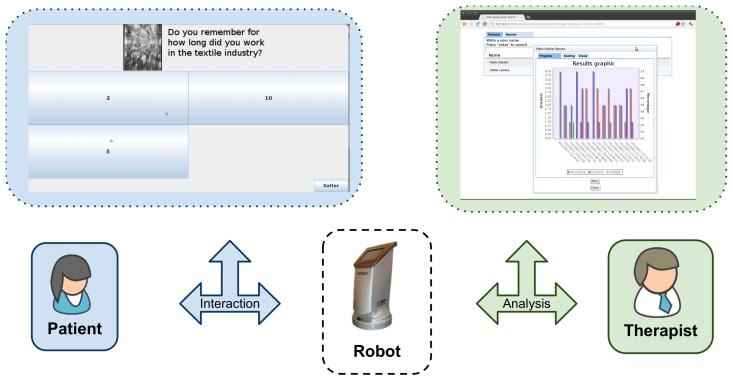
Usage diagram of the health scenario of the ACROSS project [7].

Figure 2 summarizes the described interaction and the existing roles. However, the robot movement in the first application was controlled by a human. A target scenario aimed to use the robot in a group of patients in a room and let the robot move autonomously from one patient to another, which is already a challenge. Indeed, the next logical step would be moving in the health center from one room to another or even going with the patient to the room. This could even be scheduled by the therapist once a day, where the therapist details to which rooms the robots should go at which times. All these types of movements become a special problem that must take into account the patient's speed and problems that arise in the context.

Additionally, the inputs and outputs of the robot may need to be configured to enhance the user experience. For example, the level of sound depends not only on the person but also on the room where the robot resides. During the experimental session, the patient had problems pressing the tactile buttons, since she pressed them for longer than regular users, so the application should also configure itself for that type of user. In summary, as detailed in the introduction, a high level of flexibility was desirable in the scenario.

## 3. Robot Challenges in a Healthcare Environment

The application requirements explained in Section 2 can be enumerated from the point of view of robotics as follows:

1. **Flexibility in human-robot interaction:** in the case of the target scenario, the flexibility in interaction is not only convenient, but needed. Due to the varied requirements of the users, interaction modes should be adapted to the users' profile. Flexibility affects, for example, the usage of the touch-screen (changing font sizes or accepting longer tactile button pressing times), the volume of the speakers, or how the messages are communicated.
2. **Flexibility in task scheduling and execution:** depending on the status of the robot hardware and the monitoring of the task execution, the robot should be able to re-plan how to proceed. For example: abort a patient guiding task when batteries are too low, give indications if a task has been aborted or change the scheduling when a task is taking too long.
3. **Flexibility in resource management:** resources' usage should not be static and pre-defined. The robot should be able to change its resources' configuration depending on the contextual information, switching all the interaction devices to stand-by if the battery is low and interaction devices are not needed, disabling navigation abilities if motors report an error, and so on.
4. **Flexibility in software configuration:** many of the software components running in the robot are highly dependent on the parameter values used. Those parameters can change some behaviors quite significantly, making them more suitable for different situations. Good examples of the expected flexibility in software configuration are slowing down navigation speed if a patient is a disabled person or changing obstacle avoidance behavior if the robot is surrounded.

Those requirements can be met using **self-configuration** approaches. The robot, depending on the **information extracted from its context** and the **models** of the environment and itself, can **take decisions autonomously** about the configuration of its resources and services.

This paper presents a novel approach for robot self-configuration based on rule-extended ontological reasoning. Ontologies allow to model symbolic information of the environment and the robot itself. Some advantages of ontologies are that they enable reusability, they allow to model not only the concepts but the relations among the concepts, and many reasoners can work on top of ontologies.

A reasoner is used to infer new information from the ontology and take advantage of the implicit rules of relations among concepts. The reasoner works also as a rule engine, where human knowledge can be encoded as rules. Those rules are the policies the robot uses to take decisions about the best configuration of services and resources depending on the situation modeled in the ontology. Besides, rules have many practical benefits, such as flexibility to change them dynamically avoiding to re-compile the application and its logic, or the restrictions defined in rule engines that add consistency to the whole reasoning system.

As the reasoner has to be continuously updated with new information, a link between robot sensor-actuator and the reasoner is needed. This link is a robotic framework that makes possible to integrate sensor and actuator drivers with higher-level robot skills such as navigation or feature extraction. The framework selected for this paper is Robot Operating System (ROS) (see Section 3.2). ROS allows the robot to grab information from the context, to update the reasoner and apply the configuration changes inferred by the reasoner to the robot.

### 3.1. Semantic Reasoning Challenges

The proposed system uses a semantic reasoning component based on the triple space computing (TSC) paradigm. TSC was used in the initial scenario presented in Section 2, using the *otsopack* middleware [10], since the scenario required the distribution of information among multiple agents.

TSC is a shared memory paradigm that stores data as RDF triples. This data store, called space, allows the system to query and share information easily among the nodes spread in the environment. Additionally, the nodes can use a semantic reasoner to infer more information and populate the data store with it.

The focus of this contribution is to explore the capabilities of semantic rules in the area of self-configuration of robots, and not the distribution patterns. For this reason, for the implementation of this system no TSC implementation has been used. However, the same API has been used so as to be able to integrate these results in the future. For the same reason, the restrictions of this type of inference still apply. For instance, other types of reasoning [11] may not be applied and it would be interesting to take into account the context uncertainty and vagueness [12] so as to better model the environment.

**Figure 3.**
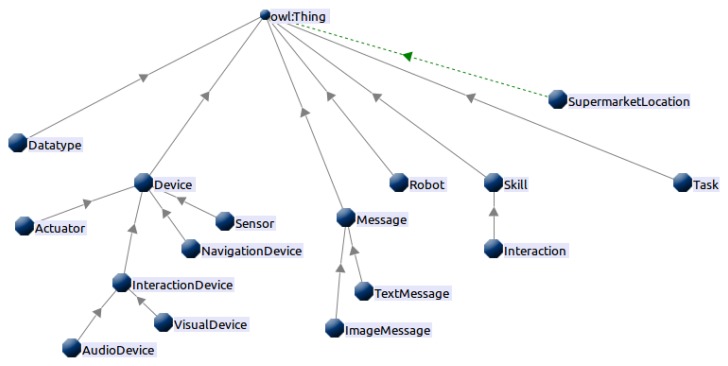
High level view of the ontology.

Due to the semantic capabilities of TSC, an ontology has been designed to represent the information relevant for the self-configuration system. The self-configuration ontology, presented in Figure 3, describes:

1. The robots' devices classified depending on their nature (sensor and/or actuator), their use (for navigation, interaction, …) and their status (unavailable devices).
2. The robots' skills (navigate, interact, …).
3. The tasks available (going to the dock, attending a patient, …).
4. The robot status (location, devices, …).

**Figure 4.**
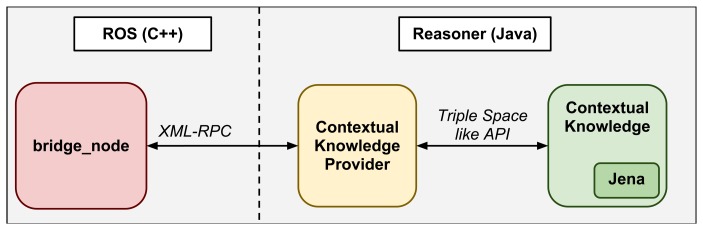
TSC components overview: the bridge node shown in 7 communicates with a XML-RPC wrapper of a TSC-like API that internally uses Jena in this implementation instead of relying on other nodes.

The aim of the described semantic component is, depending on the status of the robot, to enable/disable robot skills and to perform some task (like going to dock station to charge batteries) to guarantee its correct and autonomous operation. In order to achieve this level of reasoning, the Apache Jena semantic framework http://jena.apache.org has been deployed over this component, as shown in Figure 4.

**Figure 5.**
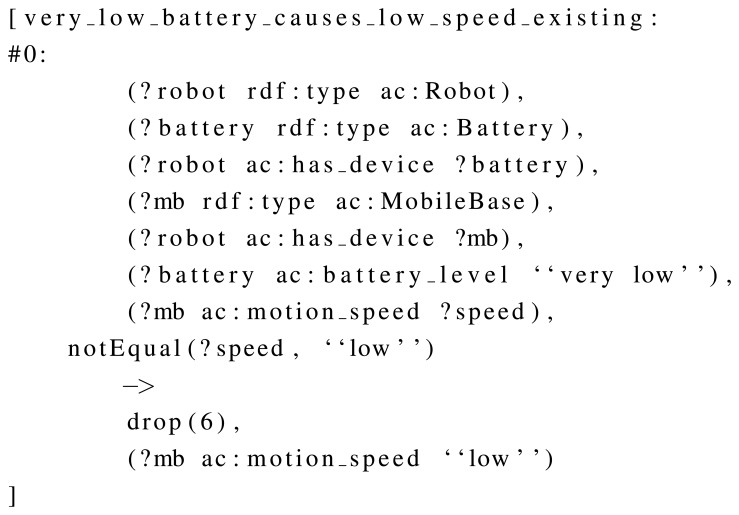
A self-configuration rule which establishes a low motion speed if the battery is low.

**Figure 6.**
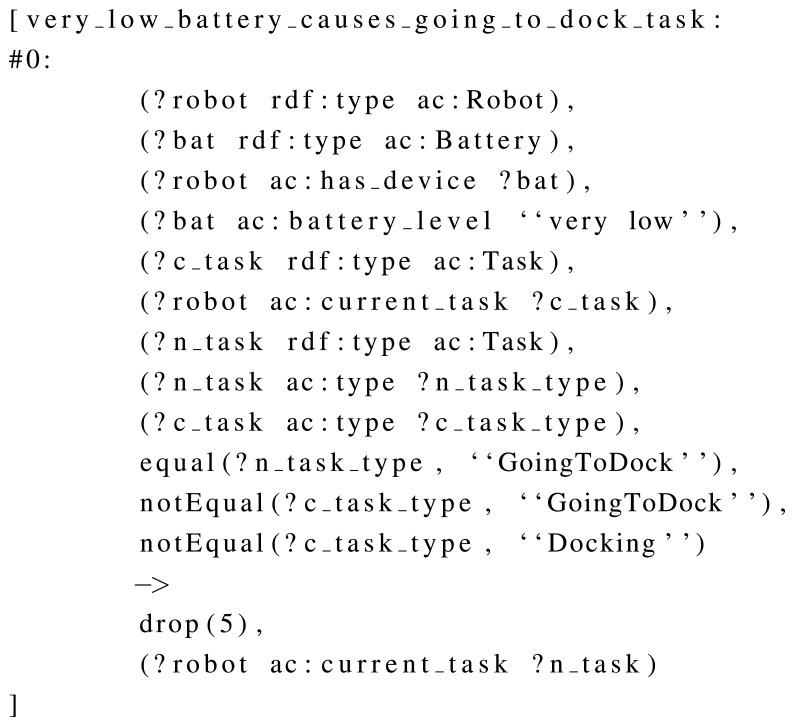
A self-configuration rule which establishes that the robot goes home if the battery level is very low.

OWL DL was used in memory, due to the requirements of the robot, and additional rules have been developed to extend the information inferred by the semantic framework, such as the one shown in Figure 5. This rule sets the *low* speed if the battery status is *very-low*. In order to do this, the rule defines a situation using the stored knowledge (e.g., “there is a robot, this robot has a battery and a mobile base, this battery has *very-low* as level, and the mobile base is not in *low* speed”), and defines an action for it. This action consists in dropping a particular triple (the speed of the mobile base) and replacing it by a new one (the mobile base must be in *low* speed). In order to show another example, Figure 6 uses the same condition (battery status being *very-low)* to bring the robot to the dock, by establishing *going-to-dock* task as current.

These levels (e.g., *very-low* battery, *low* speed) are later defined by the robot developers. Once they classify what are the levels of battery or speed, they populate the knowledge store with it. This way, they can later modify the rules adopting them to the new requirements from a high level perspective, working on abstract concepts. This enables maintainers to adapt and customize the behavior of the robot without needing to deal with the code by changing the rules files, and working on top of existing rules provided by those ontologies on top of which this one is developed. Additionally, keeping the rules abstract enough enables sharing the same rules among different types of robots.

### 3.2. Integration in ROS

ROS http://www.ros.org/ is an open-source software development framework for robots. It is presented as a meta-operating system for robots, where services such as hardware abstraction, low-level device control, implementation of commonly-used functionality, message-passing between processes, and package management are provided. ROS offers the means to develop distributed robot applications, with a peer-to-peer network of processes called *nodes* that are loosely coupled using the ROS communication infrastructure. In the recent years, ROS has become a *de-facto* standard in robotics. Due to its extensive use by robot developers, ROS has a wide repository of software components such as sensor drivers, visualization tools, navigation systems, arm manipulation systems or artificial vision algorithms. Besides, ROS is being actively supported by the *Open Source Robotics Foundation* http://www.osrfoundation.org/. Based on all those features, ROS was selected for the developments presented in this paper.

The application described in Section 2 presents several challenges that are already addressed in some of ROS stacks, which are collections of algorithms and tools with a defined objective. Here is a brief description of the stacks used in this paper and their objectives:

1. Navigation stack http://www.ros.org/wiki/navigation: to make the robot move among the places of target scenarios, autonomous navigation is required. ROS Navigation stack provides the algorithms for that purpose. From a high-level point of view, the navigation stack offers two main features to the robot: (i) the capacity to go from any point of the environment to any other point and (ii) the capacity to localize the robot with respect to the map of the environment.
2. Diagnostics stack http://www.ros.org/wiki/diagnostics: to make all the hardware status available in a standard way, the diagnostics stack is used in this paper. Diagnostics stack provides message definitions for hardware status monitoring.
3. Dynamic reconfigure http://www.ros.org/wiki/dynamic_reconfigure: this tool is used to change the parameters of ROS nodes dynamically. For example, a navigation node has a parameter to define the allowed maximum speed of the robot. With dynamic reconfigure this parameter can be changed dynamically during execution. This feature is very interesting in this paper, since parameters are changed depending on the reasoning output.

**Figure 7.**
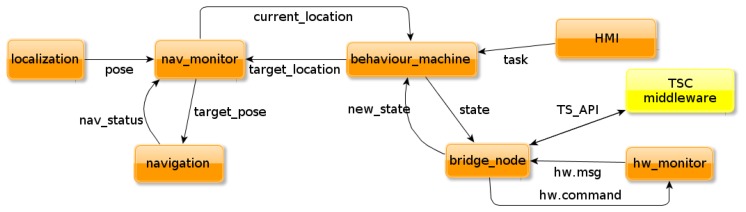
Components diagram of the ROS side and its integration with the TSC middleware.

A software architecture has been designed to integrate the TSC middleware into ROS and make good use of the described ROS stacks and tools. The architecture is depicted in Figure 7. The crucial software component or node (as called in the ROS nomenclature) is the *bridge_node*. All the communication between the ROS world and the Triple Space is centralized in this node, decoupling both systems and therefore not increasing the complexity in any of the two components. The *bridge_node* uses ROS communication mechanisms to subscribe to other ROS nodes that provide online information of the context or the robot itself. To update the Triple Spaces with context information, the *bridge_node* uses the *TS_API*. That *API*, in addition to providing the functions to set information in the space, also provides the means to notify any changes in the ontology. The *bridge_node* uses such notifiers to get the conclusions extracted by the reasoner on the new context information. Those conclusions are subsequently transmitted to suitable ROS nodes using ROS publishing mechanisms.

The software architecture for TSC middleware integration into ROS is presented in Figure 7. On this figure, orange boxes are ROS nodes while the yellow box depicts the TSC middleware; behavior machine is the node where the state machine of the robot tasks is implemented. Additional nodes refer to the hardware components of the robot, the human-machine interaction and navigation system. Let us briefly explain them:

- *HMI*: it stands for *Human Machine Interface* and it is a simple interface where a user can assign tasks to the robot
- *behavior_machine*: it is the responsible of handling the internal task automaton of the robot, depending on the inputs provided by the user and the reasoner
- *hw_monitor*: this node uses ROS diagnostics stack to monitor the status of every hardware device of the robot. It is also the responsible to command hardware drivers with the new decisions made by the reasoner and communicated via *bridge_node*. Even though the architecture does not show explicitly, this node is connected to nodes encapsulating hardware drivers (they are not shown to keep the architecture clean)
- *nav_monitor:* this node abstracts navigation functionalities. It receives a semantic target place (e.g., “kitchen”, “bedroom”…) and translates it into map metric coordinates. Additionally, it provides the information about current location (translating from metric coordinates into semantic places) and navigation status (target reached, navigating, navigation aborted)
- *navigation* and *localization*: both are part of the ROS navigation stack, providing path-planning and obstacle avoidance capabilities (*navigation*) and metric localization in a known map (*localization*)

### 3.3. Deployment of the Proposed Solution

During execution time, the proposed solution works in a continuous loop where the following steps are sequentially repeated:

1. **Extract relevant information from the context:** using on-board sensors and specific algorithms, the robot extracts relevant information from its environment and itself. The context information directly maps to the designed ontology and it is specific to the application. In the healthcare scenario, examples of such information are: robot location (patient room, hall, corridor…), current task (guiding a patient, going to dock…), current robot speed, the status of a hardware device (failure, warning, working), etc.
2. **Update the Triple Space:** every new piece of information generated by the robot is captured by the *bridge_node*, as explained in Section 3.2. Subsequently, this node updates the Triple Space with the new information available. The values of the instantiated ontology change accordingly.
3. **Process the new information and check the rules:** the reasoner, as soon as new information is set in the Triple Space, checks the rules written for the application to find which rules should be activated. Then, the reasoner executes the activated rules and the status of the instantiated ontology changes again, reflecting the conclusions extracted by the reasoner.
4. **Capture the conclusions of the reasoner:** once again, the *bridge_node* captures the changes in the instantiated ontology due to the execution of the activated rules. As shown in Figure 4, the node uses the *TS_API* for that purpose.
5. **Command the robot:** the *bridge_node* publishes the produced changes. Every ROS node connected to the *bridge_node* is subscribed to the relevant information for that node. They capture the information and act consequently, commanding the involved actuators and software components.

Let us see the whole process with a simple example. There is a rule that states that if the battery level is very low, the robot has to go to the docking station in order to recharge its battery. There is another rule that says that if the battery level is very low, robot maximum speed should be low to try to save as much energy as possible. Furthermore, there is a final rule that says that all the interaction devices have to be switched to stand-by mode, once again to try to save energy. These three rules are activated when the battery level falls to very low.

It is highly recommended to look at the Figure 7 during this example in order to understand the flow of information. The process starts in the battery driver, which is continuously monitoring the charging level. When the battery measures charging levels that go below certain threshold, the battery driver publishes a *very low* level (step 1). The *hw_monitor* node extends this information until it is grabbed by the *bridge_node*. Immediately, the *bridge_node* updates the Triple Space with the new information about battery, using the corresponding function in the *TS_API*. The instantiated ontology is thus updated with a new value for battery charging level: *very low* (step 2).

Consequently, as new information has been set, the reasoner checks all the rules of the system. It finds that there are three rules that have to be activated regarding the new value of the battery. As explained before, the first one says that robot's current task has to be switched to a “*going_to_dock*” task. Hence, the reasoner changes robot's current task's value in the instantiated ontology. The second rule is about the maximum speed for navigation, which has to be set to “*low*”. Finally, the third rule demands more work from the reasoner. The rule states that every interaction device has to be switched to stand-by mode. Using the relations defined in the ontology, the reasoner can infer that the touch-screen, the speakers and the microphone are interaction devices. However, the wheel motors and the laser scanner are not. In consequence, the reasoner only updates the “*running_mode*” of the interaction devices (step 3).

All those changes in the instantiated ontology are notified to the *bridge_node* (step 4), which publishes the new information to the subscribed ROS nodes using the communication mechanisms of ROS (step 5). For instance, the *behavior_machine* receives a new task assignment. As it comes from the *bridge_node*, the *behavior_machine* changes robot's current task, whatever it was before. One of the consequences is that the robot has to go to the docking station, so the *nav_monitor* is commanded with a new target position. This is not the only change for the navigation part, since the *nav_monitor* is also commanded to limit the navigation maximum speed.

Finally, the *hw_monitor* also receives an update from the *bridge_node*. The node receives new *running_mode* values for several devices. All the driver nodes of such devices are subscribed to *hw_monitor*. They receive the command that they have to switch to stand-by mode. Each of them will use the functionalities of the driver to obey the command.

The effect on the robot is quite clear. It will stop doing whatever it was doing and it will head towards the docking station. In its way, the robot will limit its own speed to save energy. Additionally, it will also switch all the interaction devices to stand-by, since they are not needed for its mission and they waste energy.

The example illustrates how different rules are combined to create a perfectly coherent behavior in the robot. It also shows how the process is executed online, from a hardware event that propagates itself, to well-defined and noticeable changes in the behavior and devices of the robot.

## 4. Evaluation

This section evaluates the suitability of the proposed approach in the defined scenario. Therefore, the focus of this section is not measuring the underlying semantic framework (Jena) or comparing it with other frameworks, but to check if the time required by the solution is acceptable and at which level.

In the context of the proposed scenario, there are two variables: first, the number of triples in the ontology, and second, the number of rules. The first one, however, is not subject to frequent change, given that the components available in the robot are fixed and the proposed ontology is not modeling the whole context. However, the number of rules that can be created is defined by the developers, so it may increase significantly.

**Figure 8.**
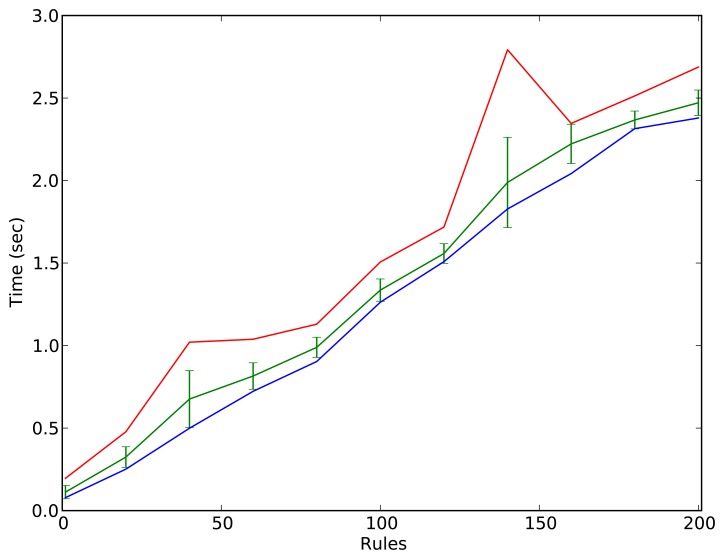
Number of rules (horizontal) and time for updating the ontology and standard deviation, expressed in seconds.

For this reason, the impact on performance by the number of rules has been analyzed. Different numbers of rules that add and destroy triples in the ontology, keeping its size stable, have been defined, from 1 to 200. In each case, 10 measurements have been taken and the trend is presented in Figure 8, showing the maximum value, the minimum value, the mean and the standard deviation. The particular data is presented in Table 1.

All the measurements have been taken in an Intel Core i7 running at 1.90 GHz, using Jena 2.6.4 and Java 7, and running Ubuntu 12.10.

To analyze the suitability of the proposed solution for robotics applications, based on the obtained results, robotics architectures must be analyzed first. It is very difficult to choose a reference architecture for robotics applications, since the research on that topic has been very intense. A good summary of the different approaches can be found in Arkin's book [13]. Broadly speaking, three main architectures can be distinguished in the robotics community: reactive, symbolic and hybrid architectures. Nowadays, it can be said that hybrid approaches are more used than the others. Indeed, the architecture presented in this paper is a hybrid architecture.

The concept of a hybrid architecture usually identifies two layers: the reactive and the deliberative layer. The first layer is designed for light and fast behaviors, where short-term decisions have to be made. This layer is usually connected to the sensors and actuators of the robot. On the other hand, the deliberative layer uses higher-level information to make decisions on the long-term. The deliberative layer uses bigger amounts of data and builds plans as sequences of short-term targets that are sent to the reactive layer. Hence, the reactive layer operates constantly at high rates (10–20 Hz depending on the specific behavior), whereas the deliberative layer is slower (∼1 Hz, depending again on specific behaviors).

An example of a hybrid architecture is the navigation system used in this paper. It has a global planner or path-planner that, having the complete map of an environment, plans the best trajectory from the robot pose to the target pose. The planner is based on deterministic heuristic based search algorithms (very similar to the popular A*). The outcome trajectory is a sequence of intermediate poses. Those poses are sent to the reactive layer or local planner that, using sensor information, computes the best velocity commands to reach the commanded poses while avoiding any obstacle of the environment. For that purpose, the local planner uses cost functions and kinematic constraints, combined with local (small) maps built from sensor data.

Looking at the results obtained in Table 1, it is obvious that the semantic framework presented in this paper cannot be used in the reactive layer. It should be used for those behaviors that do not require fast response, *i.e.*, it should be used in the deliberative layer. This result completely fits with the approach adopted from the beginning of the project, where the semantic framework was planned to implement no-time critical self-configuration behaviors. Not only the results but also the concept of the semantic framework itself fits with the deliberative layer definition, so the results are not surprising at all.

However, it is important to point out the scalability of our solution in terms of computation time. Even when testing it with 200 rules (see Table 1 and Figure 8), the response times are smaller than 2.5 s. Although it is difficult to estimate how many rules are needed to implement a concrete application (the variables are unpredictable), the ACROSS use-cases were solved using around 40–50 rules. In consequence, the response times of the framework were below one second, which is more than acceptable for a deliberative component.

In conclusion, the results obtained regarding response time clearly support the suitability of the solution proposed for the target scenarios. There are time limitations that prevent the use of the semantic framework for reactive behaviors, as expected. However, the usage of the solution in the deliberative layer of robotic applications is totally feasible.

## 5. Related Work

Self-configuration is becoming a hot topic in robotics, where changing environments and high-level task specifications demand more flexible software and hardware architectures. Self-configuration research can be focused on hardware modules and robot design [14] or on robot software architectures and services. This paper fits in the second group.

Some researchers use self-configuration approaches to make robot teams configurable while operating. Based on ideas brought from the Semantic Web Services community, Gritti *et al.* [15] present a framework for discovery and composition of functionalities, which is used to make robot ecologies self-configurable. For that purpose, the authors define a mechanism of semantic interoperability to match functionalities from heterogeneous devices according to a unified ontological classification, which is shown to work for some navigation tasks.

Those works are extended by Lundh *et al.* in [16], providing a plan-based approach to automatically generate a preferred configuration of a robot ecology given a task, environment, and set of resources.

However, self-configuration has also been studied for single-robot scenarios. Literature shows extensive work on dealing with dynamic software architectures that can self-configure depending on the task and the available resources. A good example can be found in [17] where the authors develop a self-managed robot software framework called SHAGE. The framework uses ontology-based models to describe architecture and components. On top of those models, a reconfigurer is used, which can change the software architecture depending on the selected reconfiguration pattern. Such a pattern is generated using a decision maker to find the optimal solution of reconfiguring software architecture for a situation.

A similar objective is presented in [18], which aims to learn from experience robust ways to perform high-level tasks and self-configure robots' software components in consequence. Those challenges are issued in the so-called ROBEL framework. Another example can be found in [19]. Authors adopt a semantically-based component selection mechanism in which situational information around robots is represented as critical semantic information for the service robots to select software components.

On the other hand, healthcare environments have been a widely used application scenario for service robots. The most frequent duties for service robots in hospitals are transport of goods and floor cleaning. For the first application Ozkil *et al.* [20] present an extensive survey, analyzing the benefits of introducing service robots for transportation. As a transport example, Carreira *et al.* designed a service robot to deliver meals to patients [21], whereas Ozkil *et al.* show the design of a fleet of robots for general transportation of goods [22]. Finally, a European project called IWARD http://www.iward.eu/ was devoted to introducing a self-configuring robot team for hospital cleaning, surveillance, drug delivery, environment monitoring and virtual consultancy, as described in [23].

## 6. Conclusions and Future Work

This contribution has presented a novel solution for addressing self-configuration in healthcare social robots. The impact of this contribution is two-fold, since it presents an approach that is not restricted to scenarios similar to the scenario detailed in Section 2 and because it provides a new insight for robot self-configuration, using it as a way to program social robots' complex behaviors.

The approach presented in this paper is extensible to those scenarios where social robots are involved in a complex and dynamic environment in which the robot must react. To adapt the solution to new scenarios, two areas have to be changed mainly:

1. The ontology, which has to capture the knowledge about the environment, the robot, the users and the tasks. If new semantic classes and relations are needed, it is easy to extend the ontology. The main purpose of the ontology is to model the domain of the application.
2. The rules, which have to encapsulate robot behavior with respect to the events in the scenario. Each scenario has its own suitable behaviors and criteria to cope with the diverse situations that happen during social interaction. Rules have to encapsulate human knowledge about those situations and social behaviors.

During the ACROSS project, the versatility of our approach was demonstrated. Apart from the healthcare scenario, ACROSS also had another use case in a supermarket. Briefly explained, the robot assisted supermarket clients when they were shopping. That implies guiding clients to different places to find any product, giving advice about healthy products and habits, informing about the best offers of the supermarket and similar tasks. As the skills demanded by the robot for those tasks were very similar to those needed in the healthcare scenario, adapting the ontology and the rules was a straightforward task. However, programming all those new behaviors from scratch, using for example programming languages, would be a very tough work. Our approach enables developers to use a high degree of expressivity that we believe boosts the programming of social robots.

Regarding future work, a deep evaluation in terms of flexibility when compared with the original work will be interesting. Several positive and negative factors have an impact, such as the learning curve when developers have low or no experience with semantic web and rule engines, the tools used and the development speed (e.g., IDEs will provide quicker feedback reporting an error during development than the rule engine), lines of code *versus* size of the few rules, and the number of unexpected situations covered. Our initial tests in ACROSS suggest that for experienced developers, using our approach really makes a difference in development time. Moreover, due to the high-level semantic nature of our approach, even robot users could be trained to write their own rules and behaviors, making social robots more accessible to non-expert people.

Finally, it is worth to comment that at the moment the rules are manually defined by developers. However, it would be interesting to explore the implementation of certain degree of automation process in the development of the rules, so the robot itself may learn from the situations and dynamically generate its own rules and add them to the knowledge base. Several approaches in the literature can be found about reinforcement learning or learning by demonstration for robots. They are usually more focused on learning specific skills rather than social behaviors. Applying automatic learning techniques to social behavior learning is still an open issue.

## Figures and Tables

**Table 1.** Number of rules, mean time for updating the ontology and standard deviation, expressed in seconds.

**Rules**	**Mean**	**Standard Deviation**
1	0.112	0.040
20	0.324	0.062
40	0.676	0.172
60	0.815	0.080
80	0.989	0.061
100	1.336	0.068
120	1.558	0.060
140	1.989	0.273
160	2.222	0.119
180	2.367	0.054
200	2.471	0.077

## References

[1] Bravo J., Alamán X., Riesgo T. (2006). Ubiquitous computing and ambient intelligence: New challenges for computing j. ucs special issue. J. Univ. Comput. Sci..

[2] Hervás R., Garcia-Lillo A., Bravo J. (2011). Mobile Augmented Reality Based on the Semantic Web Applied to Ambient Assisted Living.

[3] Jara A.J., Zamora M.A., Skarmeta A.F. (2011). An Internet of Things—Based personal device for diabetes therapy management in ambient assisted living (AAL). Pers. Ubiquitous Comput..

[4] Robins B., Dickerson P., Stribling P., Dautenhahn K. (2004). Robot-mediated Joint Attention in Children with Autism: A Case Study in Robot-Human Interaction.

[5] Hu J., Edsinger A., Lim Y.J., Donaldson N., Solano M., Solochek A., Marchessault R. An Advanced Medical Robotic System Augmenting Healthcare Capabilities-Robotic Nursing Assistant.

[6] Kranz M., Linner T., Ellman B., Bittner A., Roalter L. Robotic Service Cores for Ambient Assisted Living.

[7] Castillejo E., Orduna P., Laiseca X., Gómez-Goiri A., Ipina D.L., Fınez S., de Asturias Parcela P.T. Distributed Semantic Middleware for Social Robotic Services.

[8] Laiseca X., Castillejo E., Orduña P., Gómez-Goiri A., López-de Ipina D., González Aguado E. (2011). Distributed tracking system for patients with cognitive impairments. Ambient Assist. Living.

[9] Gómez-Goiri A., Ipiña D.L. On the Complementarity of Triple Spaces and the Web of Things.

[10] Gómez-Goiri A., Orduna P., Ausin D., Emaldi M., Ipina D.L. (2011). Collaboration of sensors and actuators through triple spaces. IEEE Sens..

[11] Barba J., Santofimia M.J., Dondo J., Rincón F., Sánchez F., López J.C. (2012). A reasoning hardware platform for real-time common-sense inference. Sensors.

[12] Almeida A., Ipiña D.L. (2012). Assessing ambiguity of context data in intelligent environments: Towards a more reliable context managing system. Sensors.

[13] Arkin R.C. (1998). Behavior-Based Robotics.

[14] Stoy K., Nagpal R. Self-reconfiguration Using Directed Growth.

[15] Gritti M., Broxvall M., Saffiotti A. Reactive Self-Configuration of an Ecology of Robots. http://www.aass.oru.se/~asaffio/.

[16] Lundh R., Karlsson L., Saffiotti A. Dynamic Self-Configuration of an Ecology of Robots. http://www.aass.oru.se/~asaffio/.

[17] Kim D., Park S., Jin Y., Chang H., Park Y., Ko I., Lee K., Lee J., Park Y., Lee S. Shage: A Framework for Self-Managed Robot Software.

[18] Benoit M., Guillaume I., Malik G., Felix I. Robel: Synthesizing and Controlling Complex Robust Robot Behaviors.

[19] Lee H., Choi H.J., Ko I.Y. (2005). A semantically-based software component selection mechanism for intelligent service robots. MICAI 2005: Adv. Artif. Intell..

[20] Ozkil A., Fan Z., Dawids S., Aanes H., Kristensen J.K., Christensen K.H. Service robots for hospitals: A case study of transportation tasks in a hospital.

[21] Carreira F., Canas T., Silva A., Cardeira C. I-Merc: A Mobile Robot to Deliver Meals Inside Health Services.

[22] Ozkil A.G., Dawids S., Fan Z., Srensen T. Design of a Robotic Automation System for Transportation of Goods in Hospitals.

[23] Thiel S., Habe D., Block M. Co-operative Robot Teams in a Hospital Environment.

